# Ties That Lift and Bind: How Relationships Empower and Disempower Careers

**DOI:** 10.3390/bs16030385

**Published:** 2026-03-07

**Authors:** Mirit K. Grabarski, Galina Boiarintseva, Maria Mouratidou, Hina Kalyal

**Affiliations:** 1 Faculty of Business Administration, Lakehead University, Thunder Bay, ON P7B 5E1, Canada; 2 Department of Management and Marketing, Niagara University, Vaughan, ON L4K 0K4, Canada; galinab@niagara.edu; 3 Liverpool Business School, John Moores University, Redmonds Building, Brownlow Hl, Liverpool L3 5UG, UK; m.mouratidou@ljmu.ac.uk; 4 Hill Levene School of Business, University of Regina, Regina, SK S4S 0A2, Canada; hina.kalyal@uregina.ca

**Keywords:** career development, career empowerment, relationships

## Abstract

In this research study we take a linked lives perspective and apply interdependence theory to explore how meaningful relationships impact individual career decisions over the life course. We conducted qualitative semi-structured interviews with 31 participants and analyzed the data in a thematic analysis procedure, taking a mixed approach where data was openly coded, a subset identified and isolated, classified into categories, and openly coded again. Our findings not only demonstrate how desirable and overall positive relationships can be empower individuals’ careers (supporting, mentoring), but how they can also limit and disempower them (constraining, distracting). This study reopens a discussion on the role of relationships in careers, acknowledging the positive and negative aspects they may have, thus complementing the agentic perspective that is currently dominant in career theory. We provide empiric support to ideas that are often used in career counseling, identifying the individual’s unique set of relationships and the impact they have.

## 1. Introduction

While the theories have focused more on the individual in recent decades, ideas of relational influences have always been a part of careers research (Hall, 1996b). Looking back, we can see that many career theories have embedded social aspects, showing how individuals make choices at different life stages that will fit their dominant role (Super, 1990) or needs (Sullivan & Mainiero, 2007), how opinions of important others shape one’s self-perception and self-efficacy (Lent et al., 1994), and how creating and maintaining relationships can be beneficial for one’s career (Arthur et al., 1995). Recently, relational aspects have been included in the Sustainable Careers framework (De Vos et al., 2020) as part of the context that surrounds the individual. Even though research attention shifted from system-level factors to individual autonomy and one’s responsibility for their own career development, people are not completely detached from the environment, and there is still a large degree of interdependence with many other individuals. While in the workplace, people have relationships with supervisors, colleagues, and sometimes mentors, their careers are also affected by their family of origin, current family, friends, and the wider society. The diverse set of relationships that affect a person’s career have been previously named “constellations” (Kram, 1985), which provide a context for understanding individual career choices and behaviors by grounding them in social conditions. Such constellations include combinations of multiple work and non-work connections. At work, connection to others helps us build our identities (Sluss & Ashforth, 2007), develop a sense of belonging to the workplace, and construct meaning and purpose (Blustein et al., 2004; Blustein, 2011). High-quality relationships promote flourishing at work and contribute to employee well-being and work outcomes (Dutton & Heaphy, 2003), as well as sometimes crossing boundaries, becoming personal (Chory & Horan, 2023). Work relationships also have instrumental advantages: people shape their careers through networking, i.e., building, maintaining, and using informal relationships in the context of work (Wolff & Moser, 2006). On a higher level, social capital (“knowing whom”) has become a central career competency (Inkson & Arthur, 2001), and as one’s network provides benefits such as access to information, access to resources, and career sponsorship (Seibert et al., 2001), networking behaviors that allow developing social capital are positively associated with objective and subjective career success (Forret & Dougherty, 2004; Wolff & Moser, 2009). In terms of specific relationships, there is abundant research on the role of supervisor support (Fu et al., 2025) as well as mentoring and its key importance, especially for people with disadvantages (Jones-Morales & Konrad, 2018; Kram, 1985).

Beyond relationships at work, non-work relationships have also been found to be important for career success by serving other functions apart from in-work relationships, e.g., psychosocial support (Marcinkus Murphy & Kram, 2010). One key relational circle is that of the family. The search for work–life balance often guides career choices in different stages of life, as can be seen in Super’s (1990) Life-span Life-space theory and Kaleidoscope career theory (Sullivan & Mainiero, 2007), among others. People sometimes choose to withhold career advancement in order to spend more time with their loved ones as they settle and start a family of their own. Lack of balance and support at home can create tensions that have negative consequences and affect one’s work life, while a supportive partner can create a positive spillover that will advance his or her career (Crouter, 1984). But in addition to that, the family of origin is often responsible for forming people’s career aspirations and behaviors, such as through shaping their self-perception (Lent et al., 1994; Wang & Dong, 2024), projecting norms and expectations (Fantinelli et al., 2023; Middleton & Loughead, 1993), and passing on careers as inheritance (Inkson, 2004). Finally, friends and peers also play a role in one’s career development, by providing reference points for career success (Heslin, 2005).

Because constellations usually consist of positive and desirable relationships with people who assist others in developing their careers (Higgins & Thomas, 2001), relationships are often discussed in a positive context, and lack of such relationships is assumed to be a factor in stalled career development (e.g., small or weak network, or insufficiently strong emotional ties). Negative relationships are limited to mentions of competition or abuse in the workplace, although they can be ambivalent or indifferent (Methot et al., 2017). However, even positive and desirable relationships may nevertheless become a burden that limits individual careers, by instilling expectations, demands, and distractions that hinder a person’s career rather than helping it (Kim, 2018; Sinclair et al., 2014; Righetti & Impett, 2017). Hence, the idea of constellations may represent not just multiple relationships but also multiple directions and ways these relationships can affect careers.

The role of relationships in individual careers becomes even more salient in light of changes to the world of work and mechanization, as human labor is being steered towards emotional and social aspects that cannot be fulfilled by machines (Sousa & Wilks, 2018). In the current paper, we aim to understand the role of the different relationships in the context of career empowerment, e.g., perceived career control, taking into account the positive aspects, such as advice and support, but also the limitations that connections bring with them. Our research question is this: how do various relationships impact career empowerment?

### Theoretical Framework

Career empowerment is defined as perceived control over one’s career, a set of cognitions that embodies individuals’ motivation for future action (Grabarski et al., 2025b). Within this framework, a person that believes they have control over their career is more likely to be proactive, to take steps to pursue career goals, and to initiate changes. On the other hand, a person that feels powerless in regard to his or her career is less likely to act, and any career-related steps are likely to be reactive to the environment (for example, following a layoff). Career empowerment is conceptualized as consisting of seven dimensions: competence, meaning, impact, self-determination, clarity, growth, and support (Grabarski et al., 2025b). Thus, the career support dimension is an inherent component of an individuals’ perceptions of control they have over their career, such that agency is organically linked to the presence of interdependent relationships, which can sometimes serve as assets and at other times as liabilities. How individuals perceive this impact of interdependencies is a key factor that links the agentic views of career development that have been dominant in the academic literature since the earlier 1990s, with more relational perspectives that are more typical to career counseling research as well as to the more recent theoretical frameworks. As the concept of career empowerment is positioned as the agency component of the sustainable careers framework (Grabarski et al., 2025b), it also has a relational aspect that deserves focused attention. Our aim is to unpack how relational processes operate across different levels, impacting a person’s perception of their career path, and their subjective sense of control over it.

To further expand our understanding of relationships in the context of career empowerment, we draw on interdependence theory (Kelley & Thibaut, 1978) and social interdependence theory (Johnson & Johnson, 1989), which explain how, in relationships, partners’ outcomes are interconnected and mutually dependent through ongoing patterns of interaction and exchange. From this perspective, relationships are not static sources of support or constraint but dynamic systems in which each partner’s goals, emotions, and actions continually shape the other’s experiences. Applied to careers, this framework suggests that individuals’ sense of empowerment arises not only from their own agency but also from the degree of mutual responsiveness and dependence within key relationships: for example, external encouragement can enhance one’s career agency, whereas excessive dependence or misaligned goals can diminish perceived control, such that, for both partners, individual outcomes are affected by the other partner’s actions. Positive interdependence occurs when partners’ behaviors promote mutual goal achievement, whereas negative interdependence arises when one’s actions hinder the other’s progress. This also can be applied to professional and other relationships, where cooperative interdependence can strengthen motivation and collective growth, while competitive or obstructive interdependence may erode perceived control and self-determination. Moreover, the principle of linked lives from life course theory (Elder, 1994; Elder et al., 2003) adds a temporal and developmental dimension to our understanding of relational influence. It posits that lives are lived inter-dependently and that the experiences and transitions of one individual are closely intertwined with those of significant others. In the context of careers, this means that decisions, opportunities, and turning points are not solely individual acts but occur within a network of linked lives that evolve over time. While the careers literature has recently shifted from a pure individual lens to an ecosystem approach (Baruch, 2015; Donald et al., 2024), these are broader perspectives that consider multiple actors who do not necessarily have a mutual, interdependent, relationship with each person, although some actors do (e.g., Grabarski et al., 2025a). As such, it is important to revive the conversation and to introduce a richer relational dimension to the understanding of empowerment, as interdependent relationships can be more than actors in an ecosystem. The current study aims to explore the roles of relationships beyond objective career outcomes, namely how they support or hinder subjective career control, placing agency in a context of interdependence.

## 2. Methods

The present study is based on semi-structured interviews. As part of a larger study on career empowerment, some questions were focused on aspects of perceived career control, and the question that pertains to the current study was as follows: “What are some conditions/people in your environment that you find empowering? What about disempowering?”. While there was no specific guidance to focus on relationships, this aspect emerged organically and drew our attention such that we expanded on this issue to elicit more data as the interviews continued. After obtaining IRB approval, which only allowed recruiting people at arm’s length (i.e., not personal friends, colleagues, family, etc.), we distributed a call for participants via the first author’s professional network. Those who contacted the first author were provided with the IRB-approved information letter and upon obtaining informed consent, the interview was scheduled, either in-person or over the phone. The sample size was not pre-determined, aiming to stop when no more new insights emerge, i.e., achieving saturation (Saumure & Given, 2008; Warren, 2001). However, we aimed to ensure variety in terms of employment status and occupations. As such, interviews continued until a diverse representation of gender, age, ethnic, and occupational status was achieved (e.g., blue-collar, service and managerial occupations, from early career stages to retired, and unemployed.) The total number of interviews was 31, consisting of 17 women/14 men, ages 21–71, with an average age of 47. For practical reasons, all the interviews were conducted in Canada. However, not all the participants were Canadian-born, instead originating from different countries. As such, the Canadian environment may represent a general North American context where both natives, immigrants, and temporary residents are present, and the natives themselves come from different ethnic backgrounds. All the interviews were conducted by the first author. Interviews lasted between 25 and 75 min (average length 40 min); they were audiotaped with the respondent’s permission and later transcribed. Table 1 provides the demographic characteristics of the sample.

Because this is a part of a larger study, it is important to state that the first read of the data was performed by authors 1, 3, and 4, who followed the Braun and Clarke (2006) guidelines: getting familiar with the data (initial reading); generating initial codes; searching for themes; reviewing the themes; defining and naming the themes. This initial read was performed independently, and the authors met online multiple times to discuss, compare, and make decisions regarding the coding (Saldana, 2015). This process allowed us to identify the relevant data that pertains to the theme of relationships and to focus on this subset only. After distilling the relevant data, which was based on triangulation until consensus was reached (Morrow, 2005), author 2 was invited to join the coding based on her theoretical expertise in interpersonal relationships. The data was analyzed again by authors 1, 2, and 3 independently. The first stage was data-based, where the relationships were classified into subtypes (family/partner/friends/colleagues/mentors, etc.). Then, each subtype was treated separately with open coding the different aspects of the various relationships (e.g., financial support, limitation, role modeling, and so on). The coders met online regularly to discuss their findings and to resolve disagreements.

## 3. Results

### 3.1. Non-Work Relationships

The non-work relationships that were mentioned by the interviewees referred to the current family (romantic partners, caregiving relationships, e.g., children) and family of origin (e.g., parents). They are presented in the order of the timing over the life course they were most salient: parents had more impact during early career stages and romantic partners appeared later, sometimes overlapping in time followed by the appearance of children.

#### 3.1.1. Parents (Family of Origin)

Parents emerged as enduring and deeply influential actors in participants’ career empowerment across the life course and especially in the early stages. Parental influence was often internalized early and carried forward, shaping aspirations, risk tolerance, and definitions of success long after participants achieved formal independence. For some participants, parental encouragement and pride were profoundly empowering, such as by building a sense of legitimacy and reinforcing *confidence* in pursuing a demanding and prestigious career path. P4 described how both parents actively supported her pursuit of medicine, emphasizing the pride her family expressed in her achievement:

“My parents, both my parents, especially my father, he supported that decision a lot and he just encouraged me to be a doctor… nobody else was a doctor in my family so I believe I am the first doctor in this family… everyone was basically very happy that I do this.”

Parental support also took concrete, *material* forms that reduced risk during periods of transition. P1 recounted how her parents’ financial and emotional backing made a late-career shift possible: “My mom said, ‘What would it cost? What would you need here, to live.’” Thus, the presence of a familial safety net enabled risk-taking, and parental reassurance functioned less as direction and more as permission to explore non-linear paths. Beyond financial support, extended family members provided practical assistance that directly sustained participants’ ability to combine professional and caregiving roles, especially during moments of crisis and displacement. P17 described relocating with children and emphasized her mother’s presence: “I came with two kids and my mom, that helped me through the process.” This kind of support enabled career continuity at a time when competing demands might otherwise have forced withdrawal.

At the same time, parental influence was frequently described as constraining, particularly when expectations around stability, prestige, or obedience conflicted with personal interests. P20 described internalizing parental messaging about what constituted a “valuable” career: “What I should be comes from parents’ messaging… about what is a valuable career, and for them it’s something that is financially stable, it’s secure and is socially prestigious.” These expectations created *pressure* that narrowed perceived options and shaped career decisions in ways that conflicted with personal preferences. In some cases, constraint emerged not through explicit pressure but through *emotional obligation*. P7 described remaining in a career largely out of loyalty to his father: “The reason why I stayed in my career was for the love of my dad. My ties to him as a son of a father overrode the deteriorating love for engineering.” In this case, paternal authority through emotional bonds quietly and implicitly constrained autonomy.

In later life stages parental influence shaped career direction through caregiving *obligations*. P15 described relocating to support her mother during illness: “My mother also was quite sick… she needed help around the house… so I relocated to London… helping her get comfortable in her new stage of illness.” While this decision constrained mobility, it also fostered a sense of maturity and emotional grounding that reoriented the participants priorities.

It is interesting to note that not all parental influence was accepted passively, and some participants actively resisted such influence, asserting agency. P21 recalled being urged to abandon higher education in favor of immediate, low-skilled employment: [parent said]: “Why don’t you quit [college]? You don’t need college anyway. You’re a good custodian.”…I said, ‘I don’t want that. I want a strong mind.’ … Luckily, I decided to stick it out.” This moment of resistance marked a turning point in which P21 asserted autonomy over her future, which illustrates how inherited narratives can be contested. Taken together, these accounts demonstrate that parents played a complex and enduring role in one’s career development. While parental encouragement, pride, and material support can enhance agency, parental expectations as well as emotional and practical obligations constrain it. Importantly, parental influence was often internalized, shaping career decisions in the long term, structuring aspiration, confidence, and choice across the life course.

#### 3.1.2. Partners

Romantic partners emerged as a particularly salient and emotionally charged influence on participants’ career empowerment. Unlike other familial relationships, partners were often positioned as co-decision-makers whose preferences, constraints, and expectations were tightly interwoven with participants’ career trajectories. As a result, partner relationships frequently shaped careers through both direct support and significant constraint, often simultaneously. For some participants, partners functioned as important sources of *emotional encouragement* and *practical support* that enhanced career agency. P18 described his marriage as a reciprocal partnership characterized by mutual investment, emphasizing how shared goals strengthened empowerment:

“What empowers me? Having a partner. So for [my wife] and I both that’s the thing that empowered us all the way along our career. We didn’t have anybody else to depend on. …So I backed her up in her job. She backed me up on the things that I wanted to do. She gave me the sense of freedom to take a risk, which is what I always wanted to do but never had the courage to do it, because I was just too afraid of losing my job, of not having a salary”.

However, spousal relationships could also introduce pressure to prioritize *stability* over ambition. P7 described being urged by his wife to give up on his entrepreneurial pursuits: “She said, ‘enough of this entrepreneurial business. You have to get a job.’ … I did it for her.” He framed this decision as motivated by the need to maintain the marital relationship, at the expense of career aspirations: “My fear was that if I came out hard, strong, she’s going to ditch me.” (P17). In this case, career control was negotiated away to preserve relational security.

Beyond marriage, partners shaped career trajectories through decisions about *geographic mobility*, often intertwining relational commitments with structural constraints. P1 described reshaping her international career plans to align with her partner’s employability, noting:

“My romantic partner at the time wasn’t interested in China, so I was pursuing sub-Saharan Africa… because that was somewhere where we thought he could work… His credentials were never really recognized… we made the decision that he would stay at home.”

In this situation, the participant was willing to compromise to accommodate her partner’s limited career options. However, he also made a sacrifice for practical reasons. In the light of uneven professional mobility, relational decision-making was shaped not only by the partners themselves but by broader institutional barriers, resulting in an unbalanced distribution of career sacrifice within the partnership.

Taken together, these accounts demonstrate that partners constituted dynamic sites of negotiation that includes support, compromise, and sacrifice in a specific time point. While some partnerships enhanced career agency through mutual investment, others constrained empowerment by prioritizing relational stability or requiring adaptation to structural limitations. These findings underscore the deeply interdependent nature of career empowerment, illustrating how career agency is frequently exercised with, for, and sometimes against intimate relationships.

#### 3.1.3. Caregiving Relationships

Familial relationships emerged as one of the most powerful and ambivalent influences on participants’ career empowerment. While different members—children, partners, parents, and extended kin—were often described as sources of emotional, moral, and material support, they also introduced constraints that shaped, redirected, or limited career choices, creating ongoing trade-offs. This is particularly salient in the case of children, as a central and non-negotiable factor that posed career limitations, as building their *future* requires parental presence and stability, which can overpower personal ambition. P1 explained:

“The one piece you have to make sure you never mess up is what you do with your children because you’re the only person who has that job…When I’m looking at different career options, I’ve always also thinking what’s best for me and what’s best for them… A big part of the reason I turned down a job offer that I had was that the job I have now gives me access to French private school for my children.”

When evaluating new opportunities, decision-making is inherently relational, intertwined with parental responsibility, which can result in deliberate compromises. At the same time, children function simultaneously as a source of *meaning* and not only as a constraint on mobility, orienting career choices toward broader goals, such as breaking cycles of limited opportunity. P21 explained: “All I want for my kids is to be a better version than me… I want to give them the opportunities I didn’t have.” Other participants described how the family *accepted* and accommodated them to enable career continuity, reducing conflict, albeit with an emotional cost: “My kids don’t even remember me working shift work… they didn’t necessarily need me there every night”… “I wish I was there to say good night… I wasn’t able to tuck them in every night.” (P8). Thus, even if the family is tolerant towards one’s aspirations, it often coexists with relational loss, underscoring the emotional trade-offs embedded in career empowerment. 

In this context, several participants described *intentionally* constraining their careers to preserve family stability without being pressured to do so, which is by itself an agentic choice, that in turn serves as a role model for the children. P23 stated:

“My career limitations were self-imposed… I’ve chosen not to go to the executive role yet because it meant relocation… [Years after] my daughter said, ‘I had no idea what you did or how much influence you had.’… I said, ‘You didn’t need to know, I’m still your mom first.’”

This exchange illustrates empowerment across generations, where professional identity and parental identity are balanced rather than competing. This approach frames family-first choices as legitimate and empowered rather than indicating stalled ambition: “Not all choices have to be career-wise… your family is still a choice, and they are empowered to make that choice”(P24). This reframing reclaimed agency by validating family-first decisions as *values-driven* rather than as failures. Sometimes these choices are pragmatic rather than aspirational. P27 described how parental responsibilities motivate job mobility based on obligation: “I was married when I was 18… second wife had two teenage children… one of the reasons I went from one job to another was because I needed to support them.” P25 described aligning job choices with family priorities when the family functions as an organizing principle for long-term planning:

“Now that I’m married with two young kids… my choice of jobs these days are jobs where I don’t travel a lot… I want to be home with my kids…I have a horizon of 10 years until the kids are in university… after that I could potentially stop working.”

Taken together, these accounts demonstrate that familial responsibilities profoundly shaped participants’ experiences of career empowerment. However, the framing of these choices may differ. While parenthood and family obligations can diminish agency outright, more often than not, they redefined it. While familial obligations may come at the expense of mobility or advancement, requiring accommodations and sacrifice, career empowerment may still be exercised relationally, through foresight and value alignment. Agentic choices, in this case, balance individual goals with collective well-being, aspiration with care, and ambition with responsibility.

### 3.2. Work Relationships

The work relationships that were mentioned by the interviewees referred to entities that they work with directly (supervisors, colleagues) and indirectly (the broader organization, mentors and networks beyond the current workplace). They are presented in the order of the tie strength: from immediate supervisors to broader networks. 

#### 3.2.1. Supervisors

Supervisors emerged as highly influential figures in participants’ career experiences, whose actions could rapidly enable or constrain career agency. Because of their formal authority and decision-making power, supervisors were experienced as entities that directly shape participants’ confidence, sense of control, and perceived career possibilities. Supportive supervisors that expressed *trust* and positioned their employees in roles of greater responsibility empowered them through building up their confidence. P18 captured this experience, stating: “Working with Nancy is amazing. She’s the best leader I’ve ever worked for.” In this case, supervisory support functioned as a catalyst for empowerment, encouraging greater engagement and agency in career-related actions.

Supervisors also shaped participants’ professional identity through *feedback*. Being formally entrusted with responsibility or recognized by a supervisor reinforced participants’ sense of legitimacy and competence. Conversely, when supervisory authority was enacted through criticism without support, it had a markedly disempowering effect. P12 described her experience: “She didn’t know how to provide constructive feedback, it was very critical in an openly rude and embarrassing way. Made me feel incompetent.” Thus, supervisory behavior can directly erode career empowerment, as feedback can build or undermine one’s confidence.

Moreover, because of the power imbalance inherent in supervisory relationships, negative supervisory encounters were described as particularly harmful. P5 recounted an interaction with a senior academic supervisor who had previously expressed interest in hiring her but behaved in a demeaning and sexist manner upon meeting her: “He came in, he couldn’t see me, and he said something like ‘where is that broad’… very disrespectful…So I just got up and left and I didn’t return.” This account demonstrates how a single supervisory interaction, when infused with disrespect, can abruptly terminate career opportunities. While supervisors were rarely framed as intentional adversaries, violations of *justice* expectations, e.g., incidents of insensitivity, misuse of authority, or failure to provide constructive guidance, resulted in undermined confidence. In such cases, supervisors were experienced, not as developmental figures, but as gatekeepers whose behavior restricted autonomy, and due to the asymmetry of power, employees have little room to challenge or recover from negative encounters.

Overall, supervisors played a central role in shaping participants’ experiences of career empowerment: supportive supervisors enhanced agency by validating competence and enabling opportunity, while abusive or insensitive supervisory behavior curtailed career momentum and, in some cases, prompted participants to disengage from particular career paths altogether.

#### 3.2.2. Colleagues

Colleagues emerged as an important and often ambivalent source of career empowerment. Unlike supervisors, colleagues occupied lateral positions within participants’ relational constellations, shaping careers through everyday interactions, shared norms, and informal influence rather than formal authority. Participants described colleagues as simultaneously offering belonging, support, and learning, while also creating constraints through comparison, distraction, and insularity. For some participants, colleagues functioned as a primary source of social connection and *emotional support*, particularly in careers marked by mobility or instability. P1 described how frequent relocations limited her ability to build long-term relationships outside of work, leading colleagues to become a substitute social network: “Moving around every three or four years doesn’t really allow you to develop a root system with friends and family… My own organization becomes kind of like a substitute for a family.” In this sense, colleagues enhanced career sustainability by providing continuity, belonging, and relational stability. At the same time, reliance on colleagues as a primary social circle could become constraining. While enjoying her colleagues’ company, P1 also admitted that the organization as a family substitute created a closed relational system that discouraged exploration beyond the immediate work context, thereby limiting career agency: “It’s very self-feeding, kind of devours everything, it says ‘feed to me’.”

Colleagues also influenced career trajectories through peer *norms* and shared behaviors, becoming each other’s sources of motivation, confidence, and opportunity creation. P30 highlighted how social ties with peers helped him maintain a sense of control in the face of career disruption: “I had friends everywhere… people like me and I like them.” While these relationships were not always tied to formal advancement, they provided reinforcement and resilience that supported continued engagement with work.

In summary, colleagues played a dual role in participants’ experiences of career empowerment. Peer relationships fostered belonging, emotional support, and continuity, particularly in mobile or uncertain careers. At the same time, colleagues could constrain agency by reinforcing limiting norms and narrowing perspectives. These findings underscore that collegial relationships, while often perceived as inherently positive, can simultaneously function as assets and liabilities within individuals’ career trajectories.

#### 3.2.3. Organizational Impact

Beyond interpersonal relationships, participants described the organization itself as a powerful structural context shaping their experiences of career empowerment. Organizational *cultures*, leadership climates, management systems, and institutional procedures influenced how much autonomy, innovation, and belonging individuals experienced in their careers. P12 described how exposure to toxic leadership extended to affect her relationship with the organization as a whole, “creating mistrust in organizational leadership.” This erosion of trust weakened her sense of confidence in the organization’s capacity to support her career and negatively impacted her willingness to invest in developing her career within it.

Organizational structures were also experienced as disempowering when they constrained innovation and failed to accommodate evolving skill sets. P2 pointed to the *rigidity* of traditional management systems, particularly in relation to technological change, stating: “It’s very difficult for a traditional company to do something with the Internet because they don’t have the Internet style of thinking.” In this context, outdated organizational logics limited employees’ ability to apply modern competencies, reducing perceived impact and constraining career empowerment. In addition, participants described institutional *gatekeeping* as a significant organizational barrier that limited access and autonomy, even for qualified individuals. P4 recounted an experience within an academic and professional system in which procedural hierarchies prevented direct engagement with decision-makers: “I tried to meet the residency program director, but the secretary wouldn’t even let me talk to him. You feel so small when people act like you don’t belong, even when you’re qualified.” This account illustrates how organizational procedures and hierarchical boundaries can reinforce marginalization and undermine perceived agency.

At the same time, organizations could also serve as powerful sources of empowerment by signaling *recognition* and providing tangible *opportunities*. P5 described how institutional encouragement in the form of an unsolicited scholarship shaped her educational trajectory: “I scored very, very high… and I got sponsorship money that came to me without really asking for it, so it seemed like a good idea to go to university.” This institutional recognition affirmed her competence and motivated further career progression, demonstrating how organizational systems can actively enable empowerment. Participants also highlighted how inclusive organizational cultures promoted empowerment by normalizing *flexibility* and work–family balance. P8 described her organization’s approach to parental leave, emphasizing the cultural significance of inclusive policies: “We have a lot of guys taking pat leave. I love that, that’s amazing. Yeah, parental leave.” By supporting parental leave across genders, the organization fostered a culture of inclusion that empowered employees to integrate career and family responsibilities without stigma.

Overall, organizations played a central role in shaping career empowerment by structuring access to opportunity, defining acceptable career paths, and signaling whose contributions and life choices were valued. While rigid systems, toxic leadership climates, and gatekeeping procedures constrained agency, institutional recognition and inclusive cultures expanded it. These findings underscore that career empowerment is not only relational but also deeply embedded in organizational systems and cultures that shape the conditions under which careers unfold.

#### 3.2.4. Mentors and Networks

Mentors emerged as powerful relational figures who shaped participants’ career empowerment by expanding perceived possibilities, legitimizing aspirations, and providing guidance through complex institutional landscapes. Unlike supervisors, whose influence was tied to formal authority, mentors were often described as voluntary, relationally chosen, and identity-affirming figures whose impact extended beyond immediate roles or organizations. Participants emphasized that mentorship operated through exposure, encouragement, knowledge transfer, advocacy, and, in some cases, sponsorship—often at critical turning points. For many participants, mentors functioned as *role models* who broadened their understanding of what a career could look like. P3 described encountering individuals whose work and values challenged conventional notions of success: “There were people from the workplace overseas… people who have worked in some countries where Christians are in jail… that was really inspiring to see all the different directions that people can take.” Exposure to these value-driven role models expanded this interviewee’s sense of meaningful career possibilities.

Mentors also played an important role in sustaining agency when participants encountered bureaucratic or exclusionary systems. P4 described an ongoing mentoring relationship with a professional role model who provided both reassurance and concrete *strategic guidance*: “My supervisor was actually a doctor… he was very inspirational… He said, ‘No, this can be done. You just have to confront them… Keep knocking on doors until one opens”. Several participants emphasized mentorship as a source of intellectual and professional development. P5 described a supportive professor who encouraged creative thinking: “Try and be creative, don’t just do it the way we always do it.” She also described a different mentor who translated curiosity into opportunity, noting: “He couldn’t hire me… but he could provide me with money if I wanted to do a master’s degree with him.”. Some participants described mentorship that extended into sponsorship, where mentors actively used their influence to create opportunities. P23 described learning resilience and leadership from a mentor: “how to persevere… how to stay engaged”, while also being introduced to new networks. She recounted a pivotal sponsorship moment: “She said, ‘I want to be your sponsor.’ That was a turning point.” The sponsor also provided psychological protection, framing an interview as a “meet and greet” to reduce stress. In this case, mentorship extended beyond advice to include shielding and advocacy. Long-term mentoring relationships also shaped career continuity. P25 described maintaining contact with a senior leader over time: “He moved, and I followed… multiple times he brought me into new opportunities.” Peer referrals and relational trust facilitated access to senior roles, sometimes through unexpected kinship or alumni ties.

According to other interviewees, mentorship was frequently *informal* and embedded in peer relationships when networks themselves functioned as mentoring ecosystems. P7 described how academic and peer connections expanded opportunities: “At Ivey I made some connections… networking is crazy.” P10 similarly emphasized dense social capital, explaining: “I had a huge network… through which I was volunteering for different nonprofit organizations.” These accounts highlight mentorship as relational learning rather than hierarchical instruction. For some participants access to such mentoring networks was limited due to their status as immigrants. P10 reflected: “I didn’t have any friends or family here, and I didn’t know the culture.” The loss of relational infrastructure created uncertainty and isolation, illustrating how mentorship is context-dependent.

Taken together, these findings demonstrate that mentors played a central role in shaping career empowerment by expanding horizons, legitimizing aspirations, and mobilizing access to opportunity. Whether through role modeling, advice, advocacy, sponsorship, or informal guidance, mentorship functioned as a relational resource that enabled agency within complex systems. At the same time, the absence of mentorship, or experiences of disrespect masquerading as guidance, constituted significant sources of disempowerment. Career empowerment was therefore not only a function of individual effort or institutional structure but was deeply contingent on access to mentoring relationships that translated potential into possibility.

### 3.3. Broader Social Relationships

#### 3.3.1. Peer/Friends Support and Impact

Peers and friends functioned as an ambivalent social force in participants’ career trajectories. Through informal encouragement and shared experience, peers could bolster confidence and expand aspirations, yet the same relationships sometimes reinforced complacency or constrained forward movement. Operating laterally, peer influence shaped career agency through normalization rather than directive control. For many participants, peers played an important role in fostering *self-confidence* during periods of uncertainty. P3 stated: “I think they’re the ones who shaped my decision the most, just being around answering all the questions.” Similarly, P10 described friends as emotional validators during career transitions: “A couple of friends… ‘you’re doing good and you didn’t have experience but still, you did really well.’” This encouragement reinforced self-efficacy and supported the development of a positive professional identity. In some cases, the presence of a broadly supportive peer environment created an empowering climate in itself.

Peers also shaped aspirations by *modeling* alternative possibilities of educational or professional progression. P13 described how exposure to ambitious friends in high school reframed expectations: “When I went to high school… I started to meet friends… where it was just given that you would continue on your education.” P5 recalled how a close friend “really helped me prepare for exams, how to study for them, how to prepare for them.” In these cases, peers functioned as informal role models, transferring skills and norms that expanded perceived possibilities. Peers also offered *practical advice* that redirected career choices and protected participants from costly paths. P7 described a friend working in consulting who cautioned him against pursuing a high-burnout trajectory: “You could be up and out in two years, burnt out… so why do you want to do that?” This intervention prompted more reflective decision-making and highlighted how peers can act as reality checks grounded in lived experience. Finally, informal peer networks frequently operated as access points to *opportunity*, by providing insider knowledge, referrals, and credibility. P10 noted: “I had a huge network… through which I was volunteering for different nonprofit organizations.” These accounts illustrate how peer relationships can function as social capital, enabling mobility through trust and informal endorsement.

However, peer influence was not uniformly empowering. Some participants described peer groups that normalized *disengagement*, discouraged ambition and delayed professional development. P11 reflected on an early peer environment that fostered complacency: “We were as a group just lazy… whatever happens, happens.” Similarly, P14 shared: “My bad for not really thinking what went down the road, but you’re a teenager and you really think about these things unless you’re prompted, and I was never prompted, so…”. P21 described navigating two contrasting peer groups-one associated with risk and trouble, the other with positive affiliation: “I kept at a further distance and got more involved with sports.” This account highlights peer influence as a site of both risk and empowerment, contingent on selective affiliation.

Taken together, these accounts demonstrate that peers and friends played a multifaceted role in shaping career empowerment. Through encouragement, modeling, advice, access, and belonging, peers expanded confidence and opportunity. At the same time, disengaged or risk-oriented peer environments constrained ambition and delayed progress. Career empowerment therefore emerged not only from individual effort or institutional support but also from the social worlds in which participants were embedded, worlds that could either amplify or dampen agency depending on their norms, values, and composition.

#### 3.3.2. Broader Societal Impact

Beyond families, workplaces, and organizational systems, participants described broader societal norms and expectations as shaping their experiences of career empowerment in more diffuse but deeply consequential ways. These societal forces operated as background assumptions about what constitutes a “good,” “respectable,” or “legitimate” career, often constraining agency before specific decisions were even considered. For some participants, societal pressure to uphold respectability emerged as a powerful source of disempowerment. P7 described how his career choices were shaped by the need to satisfy a wide circle of social expectations, rather than personal aspirations: “It was about making her happy, making society happy, making my father-in-law, my mother-in-law.”

Other participants emphasized how societal definitions of legitimacy quietly narrowed the range of career paths perceived as viable. P13 reflected on how certain trajectories were implicitly treated as more serious or respectable than others, shaping encouragement, recognition, and self-confidence. These societal narratives did not require explicit enforcement; instead, they operated through taken-for-granted assumptions about success, subtly constraining empowerment by quietly narrowing the range of career paths perceived as viable. P13 reflected on how certain trajectories, particularly those associated with stability, clear credentials, and recognizable status, were implicitly treated as more serious, respectable, or “real” than others. Careers in established professions with linear progression, such as finance, education, or regulated professional roles, were described as attracting affirmation and encouragement, while less conventional paths, such as creative work, portfolio careers, late educational transitions, or non-linear trajectories, were more likely to be questioned or framed as risky. P17 highlighted how societal expectations unfold across the life course, shaping not only individual choices but also how those choices are interpreted by others in the context of one’s age and life stage. These life-course expectations constrained career empowerment by narrowing the range of choices perceived as legitimate at different points in time, reinforcing conformity to established trajectories and discouraging deviation from culturally endorsed paths. Career options that aligned with socially sanctioned notions of success were pursued with greater confidence, while alternatives often required justification and were accompanied by doubt.

In summary, these accounts illustrate how broader societal forces shape career empowerment by defining respectability, legitimacy, productivity, and success. Unlike organizational rules or supervisory decisions, these influences were often invisible yet pervasive, operating through shared norms rather than formal authority. Career empowerment therefore unfolded not only within relationships and institutions but also within societal structures that quietly constrained careers choices.

## 4. Discussion

This study set out to explore the relational aspects of career empowerment (Grabarski et al., 2025b), a recent conceptualization of perceived career control. While earlier career theories emphasize autonomy, boundary crossing, and self-direction (Arthur et al., 1995; Arthur & Rousseau, 1996; Hall, 1996a), our findings reveal that career agency is routinely exercised with, through, and sometimes against others. While career empowerment by definition includes a relational component (career support), our findings suggest that the relational dimension warrants greater elaboration. We demonstrate that career empowerment, while being an individual cognition of control that acknowledges the notion of support, is shaped by a more complex relational interplay, where agency is co-constructed with others, particularly with respect to self-determination and meaning. Moreover, relationships did not simply support or hinder other empowerment dimensions; they actively reconfigured what empowerment meant in different contexts. For instance, growth (through explorations of different career options) and focus (clarifying one’s goals) were sometimes subordinated to relational stability, while relationships sometimes led to redefining meaning. This suggests that empowerment dimensions may be differentially activated or suppressed depending on relational configurations, a possibility not yet fully explored in existing models. Finally, by identifying the supportive and inhibiting aspects of relationships, this study challenges implicit normative assumptions that maximizing individual empowerment is always desirable. Instead, empowerment appeared as negotiated, and sometimes deliberately constrained as an agentic choice by itself.

In doing so, this study extends relational perspectives on careers by showing that relationships are not simply contextual inputs or supportive resources, but dynamic systems of interdependence that simultaneously enable and constrain empowerment. Participants’ careers were embedded in a dense ecosystem of various actors (Baruch, 2015; Donald et al., 2024). Despite earlier portrayals of relations as being supportive (Kram, 1985; Seibert et al., 2001), our findings describe these influences as ambivalent structures, capable of producing empowerment and disempowerment at the same time: supportive relationships frequently carried costs, obligations, or trade-offs that shaped career direction in subtle but enduring ways.

Thus, our findings link the more recent agentic career theories (Arthur et al., 1995; Arthur & Rousseau, 1996; Hall, 1996a) to classic social psychology theories, namely interdependence theory (Kelley & Thibaut, 1978) and social interdependence theory (Johnson & Johnson, 1989), reintroducing relational considerations to enrich the careers literature. It is important to note the different streams of career studies: some focused on early development (career counseling) and others focused om vocational development in adulthood, either in the workplace context or in unemployment. These separate streams lead to fragmentation of the literature, missing some relational influences and focusing on others, but not considering them at the same time. In addition, while the more recent sustainable careers framework (De Vos et al., 2020) and the sustainable ecosystems career theory (Donald et al., 2024) refer to various actors in the ecosystem, these actors can have a one-sided impact rather than a relationship. We acknowledge the importance of these influences while expanding on personal-level relationships where the sides actively interact with each other. Specifically, our findings revealed ongoing patterns of reciprocal influence, in which career decisions were negotiated within interdependent systems. Moreover, the dynamics where the same relational role (e.g., supervisor, colleague, parent) could be experienced as empowering in one context and disempowering in another suggest that empowerment is situational and interactional (based on the other sides’ actions) rather than dispositional (based on role).

Our findings also align with the life course theory’s principle of linked lives (Elder, 1994), providing critical insight into how relational influences on career empowerment unfold over time. While this theory explains how individual careers are deeply intertwined with the trajectories of others (e.g., children, partners, parents and peers), it is rarely used in contemporary career studies outside of the counseling context. We provide an opportunity to reintroduce this principle into careers research, which can benefit theory development and especially longitudinal studies that should take into account a whole-life perspective (Hirschi et al., 2020). Taken together, our findings argue for a more relational theory of career empowerment, one that recognizes careers as embedded in systems of interdependence across work, family, peer, organizational, and societal domains. Rather than viewing empowerment as an individual capacity exercised against constraints, this perspective conceptualizes empowerment as something that is produced through ongoing relational negotiations, shaped by power asymmetries, shared histories, and anticipated futures.

Our study also has practical implications: In the early stages of career development, such as in high school, it is important to recognize the key role of parents as shaping one’s self-image, expectations of a successful career, and self-confidence. While counseling literature acknowledges such influences, it may often be applied only in critical situations when a student is struggling, which may be too late. We recommend introducing the idea of career empowerment to parents of teenagers and to the teenagers themselves by school staff (e.g., teachers, school counselors) as part of career studies, rather than waiting for signs of distress. By doing so, teenagers will also develop an early awareness of the impact of future relationships and when such details will become relevant to them. Next, we recommend career professionals to address relational aspects when supporting individuals seeking help, as it may address difficulties with career decision-making. Specifically, we recommend identifying the different components of relational constellations, as influential actors may be beyond the immediate family. Finally, we also recommend workplaces to acknowledge their impact on their employees’ careers and leverage this impact towards mutual benefit by setting up mentoring relations, ensuring fairness and support in supervision, and paying attention to organizational culture.

Yet, despite the efforts to conduct a thorough study, it is not without limitations. First, as mentioned above, the interviews identified the empowering and disempowering aspects of relationships in hindsight, based on long-term career outcomes. Such retrospective analysis may be impacted by memory biases and reframing: it is possible that in the moment, what they now see as supportive was considered constraining, and vice versa. Second, as qualitative study conducted in a single North American country, the generalizability of the results is limited, despite the effort to create a varied sample. On the other hand, because of the heterogeneity concerning age, gender, occupations and ethnicity, it is not possible to know if patterns would emerge by subcategory. The subjective perceptions of the researchers play a role in interpreting the results, although measures were taken to alleviate some concerns, such as having all four authors analyze the data individually before reaching consensus. Finally, as each participant reported their own subjective outlook, we could not access the perceptions of their partners and other relationships discussed, which could potentially bring a different perspective of relational influence. Future studies should aim to include these multiple perspectives.

## Figures and Tables

**Table 1:** Demographic characteristics of the Study Participants.

Participant	Age	Gender	Current Occupation	Place of Birth	Education
1	40	F	Business-manager	Canada	Masters
2	28	F	Unemployed—not looking for a job	China	Undergrad
3	23	M	Server/student	Canada	High school
4	50	F	Physician	Pakistan	Doctor
5	71	F	Retired (social work/mental health)	Canada	Masters
6	28	F	Biology-quality assurance	Canada	Masters
7	51	M	Entrepreneur (former engineer)	India	Masters
8	46	F	Police officer	Hungary	Associate
9	55	F	Speech therapist	Canada	Masters
10	35	F	Social worker (former HR)	India	Undergrad
11	35	M	Bank teller	Korea	Undergrad
12	41	F	Financial analyst (CFO)	Canada	Masters
13	47	F	Strategic consultant/writer (former HR)	Canada	Masters
14	62	M	Restaurant owner/real estate	Canada	Diploma
15	45	F	Restaurant owner	Canada	High school
16	45	M	Manager (donations)	Canada	Masters
17	62	F	Career counselor	Bosnia	Masters
18	45	M	Writer, instructor (former IT)	Canada	Masters
19	50	F	Manager—health care (former nurse)	Canada	Masters
20	38	F	Instructor/counselor	Japan	Doctor
21	51	M	Manager—education/sports	Canada	Undergrad
22	71	M	Life coach	Canada	Masters
23	58	F	Recently retired-real estate	Canada	Masters
24	49	F	IT manager—banking industry	USA	Masters
25	51	M	Manager—operations	Israel	Masters
26	54	M	Manager—digital marketing	Canada	Masters
27	63	M	Volunteer—human rights activist	USA	Undergrad
28	21	F	Undergraduate student (health sciences)	China	High school
29	43	M	CEO—IT industry	Canada	Undergrad
30	35	M	Student—diploma (counseling)	Canada	Undergrad
31	55	M	Police officer (research)	Canada	Undergrad

## Data Availability

The data that support the findings of this study are available on request from the corresponding author.
